# The Diversity of Plant Sex Chromosomes Highlighted through Advances in Genome Sequencing

**DOI:** 10.3390/genes12030381

**Published:** 2021-03-07

**Authors:** Sarah Carey, Qingyi Yu, Alex Harkess

**Affiliations:** 1 Department of Crop, Soil, and Environmental Sciences, Auburn University, Auburn, AL 36849, USA; scarey@hudsonalpha.org; 2 HudsonAlpha Institute for Biotechnology, Huntsville, AL 35806, USA; 3 Texas A&M AgriLife Research, Texas A&M University System, Dallas, TX 75252, USA

**Keywords:** dioecy, sex determination, seed plants, bryophytes, whole-genome sequencing, two-gene model

## Abstract

For centuries, scientists have been intrigued by the origin of dioecy in plants, characterizing sex-specific development, uncovering cytological differences between the sexes, and developing theoretical models. Through the invention and continued improvements in genomic technologies, we have truly begun to unlock the genetic basis of dioecy in many species. Here we broadly review the advances in research on dioecy and sex chromosomes. We start by first discussing the early works that built the foundation for current studies and the advances in genome sequencing that have facilitated more-recent findings. We next discuss the analyses of sex chromosomes and sex-determination genes uncovered by genome sequencing. We synthesize these results to find some patterns are emerging, such as the role of duplications, the involvement of hormones in sex-determination, and support for the two-locus model for the origin of dioecy. Though across systems, there are also many novel insights into how sex chromosomes evolve, including different sex-determining genes and routes to suppressed recombination. We propose the future of research in plant sex chromosomes should involve interdisciplinary approaches, combining cutting-edge technologies with the classics to unravel the patterns that can be found across the hundreds of independent origins.

## 1. Introduction

Across land plants exists an amazing variety of strategies for sexual reproduction [1]. Species have independently evolved self-incompatibility loci [2], temporal variation in flower development [3,4], and spatial distancing of male and female organs on the same plant [5,6,7], among many others [1]. Perhaps the most extreme case is dioecy, where sex-specific structures develop on separate plants. In angiosperms, dioecy is rare, found in an estimated 5% of species, but has hundreds of independent origins across more than half of the families [5]. In the other land plant lineages, most species are dioecious, at approximately 65% of gymnosperms, 68% of liverworts, 57% of mosses, and 40% of hornworts (technically the term in bryophytes is dioicous because they are haploid when expressing gametic sex, but here we will use dioecious for simplicity) [8,9]. The frequency and phylogenetic breadth of dioecy across plants provides an unparalleled opportunity to examine the key forces involved in its repeated evolution.

Early models theorized how dioecy can evolve from a hermaphroditic ancestor [10,11,12], invoking the need for two-linked mutations: one that causes male-sterility and another female-sterility. Recombination within this region can result in offspring that are either hermaphroditic or sterile. Thus, selection is strong to suppress recombination in the region containing these two mutations, forming a sex chromosome pair. For dioecious species that express gametic sex in the diploid stage, like in seed plants, the sex chromosomes are referred to as XY or ZW depending on which is the karyotypically heterogametic sex [13,14]. In haploid-dominant plants, like bryophytes, dioecious species with genetic sex determination have UV sex chromosomes, with the inheritance of a U correlating with female gametic sex expression and a V with male [13,15]. Though some species have multiple sex chromosomes (e.g., XY_1_Y_2_ or U_1_U_2_V) [8,16,17,18], which can occur through structural changes like chromosomal fusions and fissions or through polyploidy. These differences in heterogamety and ploidy of sex chromosomes found across land plants are powerful for contrasting the evolutionary processes that impact these genomic regions, especially as the mechanisms of sex determination on sex chromosomes have now expanded beyond the classic two-locus model [19].

Here we review the recent advances in sex chromosome evolution across land plants. We start by covering a brief history of identifying dioecy and sex chromosomes, and the advances in genome sequencing that have made new discoveries possible. We next broadly review new findings in plant sex chromosomes, particularly focusing on how the sex-determining region (SDR) evolves, both with the diversity of genes that are involved in sex determination and other processes that shape these complex regions of the genome. We conclude with future directions in plant sex chromosome evolution research.

## 2. The History of Identifying Plant Sex Chromosomes

Analyses of dioecy and sex chromosomes start with the remarkable works of naturalists who, with a careful eye, characterize reproductive structures throughout development. Categorizing plants as dioecious can be traced back to Linnaeus’ *Systema Naturae* (1735), where angiosperms were classified by their floral characteristics, such as number of stamens and pistils, or by sexual condition [20]. Darwin even discussed the curiosities of dioecy in *The Different Forms of Flowers of the Same Species* (1877) [21]. In some species dioecy is easily observable. One example is hops, where female inflorescences develop the characteristic “cones” used in beer production, while males have a completely different floral architecture [22]. Another example is found in the classic dioecy model white campion (*Silene latifolia*), studied intensively since the 19th century [23], where a suite of sexually dimorphic traits is obvious at early stages of flower development. However, in other species dioecy can be more subtle. In garden asparagus, both sexes phenotypically appear similar in early stages of floral development, but ultimately the stamens degenerate in females and the ovary is non-functional in males [24]. In some species, like *Solanum appendiculatum* or kiwifruit, dioecy is even more cryptic, where females even produce pollen grains, but they are non-viable [25,26]. In non-flowering groups, like the mosses, early naturalists searched for the “hidden flowers” (reviewed in [27]), which are called antheridia and archegonia (male and female gametangia, respectively) to identify dioecious species. Antheridia are easily visible during their development, however, archegonia are more challenging to locate because they are largely enclosed in modified leaves [28,29]. It is also common in mosses to not develop gametangia [30,31,32] and disentangling individual (i.e., genetically distinct) plants from their densely grown patches can be challenging. As such, some of the first confirmations of dioecy in species like *Ceratodon purpureus* and *Bryum argenteum* were done by growing individuals from spores [30]. It is unequivocal that these kinds of taxonomic observations form the critical basis of our understanding of dioecy, in addition to other sexual systems (for databases in angiosperms see [5,33]).

Uncovering the genetic basis for sex determination began with early cytological analyses (reviewed in [34]). Dr. Nettie Stevens first discovered the correlation between the inheritance of a smaller chromosome in a meiotic pair (which we now know as the Y chromosome) with male gametic sex expression in mealworms [35]. Indeed, this clear heteromorphy between sex chromosomes was critical to their identification in further cytological studies. The first plant sex chromosomes were identified in the liverwort *Sphaerocarpos donellii* [36] and subsequently many other heteromorphic pairs were found in *Humulus*, *Rumex*, and *Silene* [16,37,38], among others [14]. However, in many plants the sex chromosomes are cytologically homomorphic, or nearly so, making identifying them through classical microscopy a challenge.

Dioecious species with sex chromosomes played a pivotal role in the modern synthesis, in particular with regard to the inheritance of sex. In the early 1900s, after the re-discovery of Mendel’s foundational work on pea plants [39,40], dioecious flowering plant *Silene latifolia* (formerly *Melandrium album*) became a cornerstone for understanding the genetic basis of sex and sex-linked traits. This is partly because it has such obvious flowers and a particularly large Y chromosome that is nearly 1.5 times the size of the X [41]. In fact, the first sex-linked gene in plants was discovered in *S. latifolia* (and the related species *Silene dioica*); the X-linked recessive lethal *angustifolia* mutation produced narrow leaves that were only found in XY male plants and never led to viable XX females [42,43,44]. Decades of irradiation studies in *S. latifolia* have been elegantly used to map deletions that lead to sex mutants [45,46,47]. Such large-scale sex chromosome irradiation experiments are still immensely useful today, and have been leveraged to map sex-determining genes on the Y chromosome in *S. latifolia* [48] and in garden asparagus [49,50].

Genomic approaches have unlocked other previously intractable analyses of plant sex chromosomes. Some of the first genome references for dioecious species include the liverwort *Marchantia polymorpha* [51], grape [52], papaya [53,54], and poplar [55], published only a few years after the first plant genome (*Arabidopsis thaliana* [56]). More than two decades later, reference genomes for over 50 dioecious species have been published (Table 1). Though there are many characteristics about sex chromosomes that have made them challenging to assemble. Due to suppressed recombination, natural selection is less effective in these regions [57,58] and they often accumulate repeats [59]. This makes assembly of large contigs using short reads improbable [60] because reads often do not span the entire repeat, causing these regions to collapse [61,62]. Linkage maps, which use recombination rates across the genome, can help pull low-contiguity assemblies into linkage groups [63], but very small sex-determining regions (SDR) (e.g., ~59 kilobases (Kb) in *Morella rubra* [64]) are hard to reliably identify and very large SDRs (e.g., >100 megabases (Mb) in *Ceratodon purpureus* [65]) are hard to put in linear order due to the inherent lack of recombination. The use of Bacterial Artificial Chromosomes (BACs) has helped to resolve some sex chromosome assemblies [66,67], but like linkage maps, this approach is labor intensive. Adding to assembly issues, sequencing the heterogametic sex in diploids can result in chimeric contigs that contain a mixture of the X and the Y (or Z and W), especially if there is low divergence between homologous regions, as is expected if suppressed recombination has recently evolved [68,69]. These issues with assembling sex chromosomes are compounded by the fact that plant genomes are overall inherently complex, with many species having high heterozygosity and abundant repeats genome-wide, in addition to frequent polyploidy [70]. Despite these complications, through much tenacity, a lot of headway has been made on plant sex chromosomes using these short-read assembly approaches.

More recently, long-read technologies, like PacBio (Menlo Park, CA, USA) and Oxford Nanopore (Oxford Science Park, Oxford, UK), have made phenomenal strides for assembling complex regions, like sex chromosomes. As the reads are on average 10–15 Kb, as opposed to 100–300 base pairs with short reads, they are better at spanning repeat regions [70,115]. Not to mention longer reads mean fewer pieces of the genomic puzzle need to be put together. Although depending on the size and complexity of the genome, even with long reads, the assembly may not be pulled into pseudomolecules and may still contain misjoins. However, in these cases, with the addition of chromatin conformation data, like Hi-C, which does not rely on linkage, genomes can now readily be assembled to chromosome-scale, including the sex chromosomes [116,117,118]. Indeed, the telomere-to-telomere, gapless assembly of a human X chromosome, including the centromeres, [119] represents the future (or really the present) for genome assembly.

The most-recent improvements in long-read technologies (e.g., PacBio HiFi), including the lower error rates, and novel computational tools for assembling these data (e.g., HiCanu and HiFiAsm [120,121]), mean phasing the sex chromosomes in the heterogametic sex may now be possible. Though there are also downsides to long-read technologies, the foremost is the requisite high-molecular weight DNA, which ideally comes from fresh, young, flash-frozen tissue. This inherently precludes the incredible taxonomic resources maintained in herbaria, as well as any other avenues that could cause DNA degradation. As such, one of the biggest bottlenecks for genomic studies of most taxa today is identifying viable (and properly permitted) tissue that can be used for the genome reference, gene annotation, and maintained for future studies.

Critical for analyses in sex chromosomes, is identifying the non-recombining SDR. Currently, a combination of both long and short-read technologies is best suited for high-quality assemblies that include the sex chromosome pair. Long reads are ideal for assembling genomes into fewer contigs, while short reads are still valuable for genome polishing (even with lower error rates in long reads; e.g., with Racon [122]), Hi-C data for additional genome scaffolding (e.g., with JUICER [117]), genome annotation (e.g., with BRAKER2 [123]), and identifying the SDR (reviewed in [124]), in addition to gene expression analyses [70]. In fact, genes annotated to the SDR that have sex-specific expression are strong candidates for being involved in sex determination.

## 3. Advances in Sex-Determination Gene Identification

### 3.1. Yam

Most species in the genus *Dioscorea* (Dioscoreaceae) are dioecious [125] and have XY sex chromosomes [81,126,127], suggesting dioecy may have evolved ~80 million years ago (MYA) [128]. In *D. alata* a recent genetic map uncovered a ~10 Mb male-specific region of the Y (MSY) [81]. However, in *D. rotundata*, data support a ZW system with a small SDR (~161 Kb) [82], suggesting a recent turnover in this species. A candidate list of floral genes has been developed in *D. rotundata* [129], but more in-depth analyses are needed to identify those involved in sex determination.

### 3.2. Asparagus

**Figure 1:**
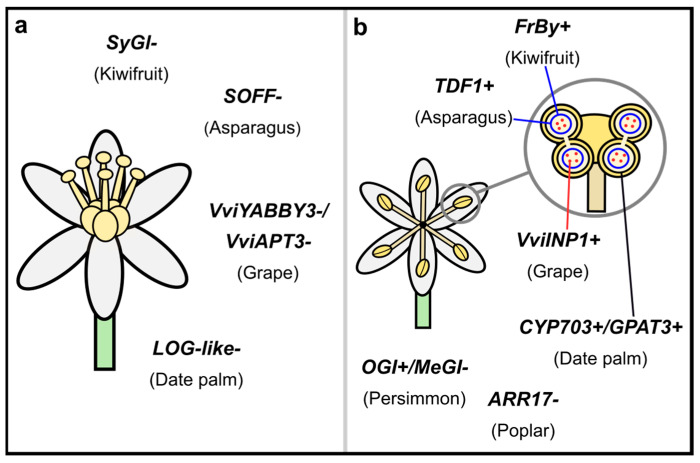
Recently discovered angiosperm sex-determination genes. Sex-determining genes recently identified that are involved with carpel development (**a**) include *SyGl*, *SOFF*, and *LOG-like*. When these genes are expressed (+) in males, it suppresses the function or development of the carpel. However, the lack of expression (-) in females allows for functional carpel development. In grapes, it is not yet known whether *VviYABBY3 or VviAPT3* is the female-sterility gene. Several genes have also been identified for promoting stamen function (**b**). *FrBy* and *TDF1* both promote tapetum development (in blue) and *VviINP1* promotes pollen development (in red). It is unknown yet whether *CYP703* or *GPAT3* is the male-determining gene in date palm, but both are involved in pollen and/or anther development. In persimmon and poplar, a single gene is involved in sex determination (*OGI* and *ARR17*, respectively). When *MeGI* is expressed, flowers develop functional carpels, but not stamens. However, when the Y-linked *OGI* is expressed, it represses *MeGI*, resulting in functional stamens. Similarly, in poplars, *ARR17* expression results in carpel production, but the lack of expression results in functional stamens.

Several species of *Asparagus* (Asparagaceae) are dioecious including garden asparagus (*A. officinalis*) [130]. *Asparagus officinalis* has XY sex chromosomes, with a ~1 Mb MSY [49] that contains 13 genes with no homologs on the X (and only one X-specific gene), suggesting suppressed recombination is through a deletion on the X [49,50]. Two of the Y-linked genes have functionally been shown to be involved in the development of the sexes through gamma ray and Ethyl methanesulfonate (EMS) mutagenesis in XY males (Figure 1) [50]. Knockouts of Suppressor of Female Function (*SOFF*), which contains a *DUF247* domain, develop hermaphroditic flowers with both functional anthers and ovules [50]. Knockouts of Tapetal Development and Function 1 (*TDF1*), an *R2R3-MYB*, make sterile individuals where neither functional carpels nor stamens develop [50]. Furthermore, knockouts of both *SOFF* and *TDF1* develop functional ovaries, but non-functional anthers [50]. Together these results show that *SOFF* and *TDF1* are the female and male-sterility genes, respectively, in *A. officinalis*. Further comparative analyses will uncover whether this sex-determination mechanism is shared across the other dioecious species in *Asparagus* or if other genes are involved.

### 3.3. Date Palm

In the genus *Phoenix* (Arecaceae), phylogenetic analyses of a *MYB1* gene suggest the XY sex chromosomes may have an ancient origin, prior to the diversification of the species in this genus [131,132]. In the date palm, *P. dactylifera,* the MSY is ~13 Mb [133,134]. Comparative analyses across all 14 species of the genus identified three potential sex-determining genes [83]. Y-linked Cytochrome P450 (*CYP703*) and glycerol-3-phosphate acyltransferase 6-like (*GPAT3*) genes are expressed only in male flowers and are likely critical for pollen and/or anther development (Figure 1) [83]. The third gene, a Y-linked, Lonely Guy-like gene (*LOG*-like), which is involved in the activation of cytokinins, is also largely expressed in male flowers, and may have a role in suppressing carpel development [83]. While these genes seem like ideal candidates for sex determination, functional follow ups are necessary to validate these putative roles in *Phoenix*.

### 3.4. Grape

All wild species of *Vitis* (Vitaceae) are dioecious. However, like papaya (described below) domestic grapes have transitioned back to hermaphroditism [135,136]. Males are the heterogametic sex in *Vitis* and in *V. vinifera sylvestris* the MSY is small at ~150 Kb and contains 20 genes [137,138]. More recent analyses show grapes also support a two-gene model of sex determination (Figure 1). The gene inaperaturate pollen1 (*VviINP1*) likely plays a role in pollen aperture formation [139] and thus male fertility. Two strong candidates for the female-sterility gene are a *YABBY3* gene (*VviYABBY3*) and an adenine phosphoribosyltransferase (*APT*) gene (*VviAPT3*) [84,140]. *YABBY3* genes have been shown to play a role in flower and lateral organ development [141] and *APT* genes are involved in the cytokinin pathway and may be involved in suppressing carpel development [140,142]. However, functional follow-ups are necessary to confirm these roles in grapes.

### 3.5. Poplar

Nearly all species in *Populus* (Salicaceae) are dioecious [143,144] and across the genera, both XY (*P. deltoides*, *P. euphratica*, and *P. tremula*) and ZW (*P. alba*) sex chromosomes have been identified, suggesting at least one turnover event has occurred [145]. In *P. tremula* the MSY is ~1.5 Mb and contains a type-A cytokinin response regulator (*RR*), homologous to Arabidopsis *RR* 17 (*ARR17*), that is found in inverted repeats [88]. CRISPR knockouts of *ARR17* in karyotypic females developed functional stamens and mostly did not develop carpels, whereas in karyotypic males, *ARR17* knockouts showed no difference in development [88] (Figure 1). Some evidence suggests gene silencing of *ARR17* in males is through RNA-directed DNA methylation, but this has not formally been tested [88]. In *P. alba*, the W also contains *ARR17* that is lacking from the Z. This intriguing result highlights how a single gene can determine sex on both diploid sex chromosome types. Although, interestingly, within the same genus, there is recent evidence of two genes involved in sex determination. In *P. deltoides* one of the sex-determining genes is also related to *ARR17,* though they call it female-specifically expressed *RESPONSE REGULATOR* (*FERR*) [146]. The ~300 Kb MSY has a duplication of *FERR* that represses it (*FERR-R*), inhibiting carpel development. The second gene, a male-specific lncRNA (*MSL*), is likely involved in promoting male function [146].

### 3.6. Willow

The genus *Salix* is sister to poplars in the Salicaceae family and most species are also dioecious [144]. *Salix purpurea* and *S. viminalis* both have a ZW sex-determination system that share an evolutionary origin having arisen ~8.6 MYA [69,94]. The *S. purpurea* female-specific region of the W (FSW) is ~6.8 Mb and interestingly contains palindromic repeats, similar to those found in humans [94,147]. Within these repeats are five genes that may be associated with sex determination. The cytokinin *RR* is particularly of interest as this gene is homologous with the sex-determining *ARR17* gene in poplar [88,94]. The *S. viminalis* FSW (~3.1 Mb) also contains *ARR17*, further supporting the putative role of this cytokinin-related gene in sex determination in willows and poplars, although this has not yet been confirmed with functional analyses in *Salix* [69]. Interestingly, >100 additional genes are found on the *S. viminalis* FSW, which show evidence of two strata. However, there is no evidence of chromosomal inversions involved in their capture into the SDR, suggesting instead the buildup of transposable elements may be involved in suppressing recombination [69]. *Salix nigra*, contrastingly, has XY sex chromosomes with a ~2 Mb MSY on a different chromosome than in the other *Salix* species examined, suggesting a translocation of the SDR (i.e., turnover) [148]. Though with current analyses it is unclear if *RR* is also sex-linked in this species [148]. Given the many turnovers and changes in heterogamety found in Salicaceae, often involving the same *RR* gene, a general model has been developed to explain this pattern [145]. Consistent with results described in Müller [88], in species with ZW sex chromosomes, *RR* acts as a dominant female promotor, but in XY systems *RR* duplicates target and repress *RR* by RNA-directed DNA methylation [145].

### 3.7. Strawberry

In *Fragaria* (Rosaceae) several species are dioecious, octoploids that are nested within a diploid, hermaphroditic clade [149], highlighting the role polyploidy can play in the evolution of dioecy [150]. Strawberries have ZW sex chromosomes that arose ~1 MYA [151]. In *F. chiloensis* the FSW is small at ~280 Kb [152], though in other *Fragaria* there is evidence the SDR is in different locations, suggesting either independent evolutions or translocations [153]. Recent evidence supports the latter, where the FSW has translocated at least twice among homeologous chromosomes, each time capturing more DNA into the region of suppressed recombination [154]. In *F. virginiana* ssp. *virginiana*, which has the smallest SDR, there are two genes, a GDP-mannose 3,5 epimerase 2 gene and a 60S ribosomal protein P0 [154]. These two genes are also located in the *F. virginiana* ssp. *platypetala* and *F. chiloensis* SDRs [154], although functional analyses will highlight whether they play a role in sex determination across these species.

### 3.8. Red Bayberry

In the genus *Morella* (Myricaceae), most species are dioecious, including *M. rubra*, the red bayberry [155]. Recent genome sequencing found *M. rubra* has ZW sex chromosomes with a ~59 Kb FSW that contains seven genes. Three of these have putative roles in flower development (Mr*CKA2*, Mr*ASP2*, Mr*FT2*) and two are related to hormones (Mr*CPS2*, Mr*SAUR2*; [64]). More functional work will help uncover which are involved in sex determination. All genes in the FSW have a paralogous copy on the same chromosome, suggesting gene duplication may have also played a role in the evolution of the sex chromosomes in this species [64].

### 3.9. Papaya

Papaya (*Carica papaya*) is the sole species in the genus *Carica* (Caricaceae) [156]. Across Caricaceae, 32 species are dioecious, two are trioecious, and one is monoecious [14]. Multiple lines of evidence suggested that sex chromosomes have evolved multiple times independently in Caricaceae, and sex chromosomes in *Carica* and *Vasconcellea* may have originated from the same ancestral autosomes after the divergence of these two genera [157,158]. Papaya is one of the two trioecious species, and sex determination of papaya is controlled by an XY system with two slightly different Y chromosomes, a MSY and a hermaphrodite-specific Y^h^ [159]. The papaya MSY is 8.1 Mb [67,160] and two large inversions in the Y-linked region caused recombination suppression with the X and initiated sex chromosome evolution [67]. No hermaphrodite papayas have been found in wild populations and the Y^h^ chromosome exhibits lower nucleotide diversity than the Y, suggesting that hermaphrodite papaya is likely a product of human domestication [136]. Several candidate genes showing functional and/or structural association with sex types were identified based on sequence comparison and gene expression analysis [161,162]. Further functional validation of candidate genes is still needed, although several independent studies point towards SVP (SHORT VEGETATIVE PHASE) as being involved in male flower development [163,164], though this putative gene does not have a sex-related function in other species.

### 3.10. Palmer Amaranth

Most species are monoecious in the genus *Amaranthus* (Amaranthaceae), however, dioecy is thought to have evolved multiple times independently [165]. The recent genome sequences of *A. palmerii* identified an XY sex chromosome system with a ~1.3–2 Mb MSY containing 121 gene models [107,108,166]. *Amaranthus tuberculatus* has a larger MSY (~4.6 Mb) with 147 genes [108]. Despite being in separate dioecious clades [165], two genes are found in the MSY of both species (Disintegrin and metalloproteinase domain-containing protein 9, *ADAM9*, and *FLOWERING LOCUS T*, *FT*) [108], making them candidates for sex determination or male-specific development.

### 3.11. Spinach

All three species of *Spinacia* (Amaranthaceae) are dioecious, and though *S. oleracea* and *S. tetrandra* diverged ~5.7 MYA, analyses of sex-linked homologs suggest suppressed recombination occurred after their divergence [167]. Recent analyses in *S. oleracea* have found the SDR to be between 10–19 Mb, with a 10 Mb MSY that has 210 genes [109,168]. These genes have been captured into the region of suppressed recombination through chromosomal inversions, making two strata of divergence between the X and the Y [109]. The 12 MSY genes with putative floral functions [109] and additional transcriptomic analyses of female and male flowers [169] have narrowed in potential sex-determining genes, though none so far are clear candidates.

### 3.12. Persimmon

Most species in Ebenaceae are dioecious including *Diospyros* [170]. *Diospyros lotus* has XY sex chromosomes with a ~1.3 Mb MSY [111]. Expression of an autosomal HD-Zip1 family gene, Male Growth Inhibitor (*MeGI*), results in the development of female flowers, with functional carpels, but not functional stamens. However, a Y-linked pseudogene, *Oppressor of MeGI* (*OGI*), encodes a small RNA that suppresses *MeGI*, resulting in male flowers [171] (Figure 1). Moreover, the male-determining role of *OGI* is stable in the hexaploid persimmon, *D. kaki*, which has both monoecious and female flowers [172,173]. These data, like in poplar, support that a single gene is involved in sex-determination in persimmons. This sex-determination system evolved through a recent whole-genome duplication, making two copies of *MeGI*. Functional analyses of these genes in tobacco suggests *SiMeGI* (sister copy of *MeGI*) may have maintained the original gene function, while *MeGI* neofunctionalized as a repressor of anther development [111]. A second duplication of *MeGI* resulted in the Y-linked *OGI*.

### 3.13. Kiwifruit

Most species in *Actinidia* (Actinidiaceae) are dioecious [174] and the sex chromosomes arose ~20 MYA [175]. Although kiwifruit is in a different family than persimmons, they are in the same order (Ericales), representing at least two independent origins of sex chromosomes. *Actinidia chinensis* var. *chinensis* have XY sex chromosomes and the MSY is ~0.8 Mb, containing 30 genes [176]. Two of these have been identified as sex determining, additionally supporting the two-locus model for the evolution of dioecy. One gene, a type-C cytokinin *RR*, suppresses ovary formation (*SyGl*), and the other has a fasciclin domain that contributes to tapetum degradation resulting in male fertility (*FrBy*) [175,176] (Figure 1). The function of these genes was validated through several approaches [176]. First, analyses of the genome of the hermaphroditic species, *A. deliciosa*, showed no evidence of a copy of *SyGl*, but did have *FrBy* [176]. This suggests either the loss of *SyGl* or the gain of *FrBy* caused the transition to hermaphroditism [176]. Moreover, knock-ins of *FrBy* into an XX female were hermaphroditic, with both functional carpels and stamens that produced fertile seeds after self-pollination [176]. Current work is in progress to also functionally validate *SyGl* [177].

### 3.14. Solanum

Dioecy evolved at least four times across the genus *Solanum* (Solanaceae) [178]. In *S. appendiculatum*, the XY system arose <4 MYA [179] and the MSY contains at least 20 genes [114]. Consistent with female flowers producing inaperturate pollen, many sex-biased and Y-linked genes are involved in pectin development [114], though more analyses will undoubtedly uncover genes involved in sex determination.

### 3.15. Amborella

*Amborella trichopoda* is a monotypic species in Amborellaceae that is sister to the rest of flowering plants [80,180]. Although the *Amborella* lineage diverged from the rest of angiosperms ~200 MYA [181], the ZW sex chromosomes are estimated to have diverged 9.5 to 14.5 MYA [182]. This recent origin of *A. trichopoda* sex chromosomes is consistent with the ancestral flower of all angiosperms being reconstructed as hermaphroditic [183]. The FSW is ~4 Mb and has ~150 genes [182], though which are involved in sex determination is unknown.

### 3.16. Maidenhair Tree

The dioecious gymnosperm *Ginkgo biloba* (Ginkgoaceae) [184] is a monotypic species. Two recent genomes suggest *Ginkgo* has an XY system [77,78] that arose ~14 MYA [77]. The MSY is ~27 Mb, with 241 genes, including 4 MADs-box genes expressed in staminate (male) cones [78]. Given the clear role MADs-box genes play in flower development in angiosperms [185], these genes are interesting candidates for sex-determination in *Ginkgo* as well.

### 3.17. Fire Moss

The moss *Ceratodon purpureus* (Ditrichaceae) UV sex chromosomes provide an interesting contrast to the XY/ZW systems in seed plants. The *C. purpureus* U and V are large with each >100 Mb and have >3400 annotated genes, totaling ~30% of the 360 Mb genome and ~12% of the gene content [65]. The moss sex chromosomes evolved at least 300 MYA in the ancestor to ~95% of extant mosses, making them among the oldest known sex chromosomes across Eukarya [65]. Compared to angiosperms, much less is known about the functions of genes in bryophytes, so narrowing in on candidate sex determiners is a challenge. However, some genes have been identified that are potentially of interest in sex-specific development. For example, the *C. purpureus* female-specific U chromosome contains an *RWP-RK* transcription factor [65], which are involved in egg cell formation across land plants [186,187] and in the same gene family as the *MID* mating-type loci in green algae [188]. Other notable genes on the *C. purpureus* U and V [65] are orthologs to the cis-acting sexual dimorphism switch found in *Marchantia polymorpha* (described below; [189]).

### 3.18. Common Liverwort

The liverwort *M. polymorpha* (Marchantiaceae) also has a UV sex-determination system with an ancestral origin [65,74]. The male-specific V is ~7.5 Mb and the female-specific U ~4.3 Mb, with 247 and 74 genes annotated, respectively [74,108], though the U has not been fully assembled, which may explain some of the difference in size. Like *C. purpureus*, it is unclear which genes on the U or V are involved in sex determination in *M. polymorpha*. However, intriguingly, an autosomal *MYB* transcription factor has a clear role in sex-specific development. Expression of *FEMALE GAMETOPHYTE MYB* (Mp*FGMYB*) results in archegonia development, whereas expression of its cis-acting antisense gene suppresses Mp*FGMYB* resulting in antheridia development and sperm production, though the sperm lack motility [189]. Several other dioecious bryophyte genomes have recently been published or are in progress [71,73,76,190], commencing an era for comparative analyses to uncover sex determination and further insights on sex chromosomes in this predominantly dioecious clade.

## 4. The Diversity of Proposed Mechanisms of Sex Determination

The plant sex chromosomes analyzed to date vary in age, size, and overall gene content, but what may be most striking is how many different genes have evolved to be the sex-determiners (Figure 1). This stands in contrast to animal systems where the same gene(s) have been shown to be involved in sex determination across many taxa (e.g., *SRY*/*SOX3; DRMT1* [191]). For the genes identified in plants, some necessary similarities exist: they must be involved at some stage of sex-specific structure development (e.g., anther or carpel). Whether certain genes in these developmental pathways are more likely to evolve sex determination than others is unknown. Genes with broad-expression patterns seem to be unlikely candidates, as sex-linkage, and any subsequent molecular evolutionary consequences like protein evolution, may be deleterious to other functions. Although, duplications, whether by doubling of the whole genome or single genes, free genes from such constraints, allowing for neofunctionalization [150]. In fact a common theme in recent studies has been that duplications play a role in sex-determining genes (e.g., *Asparagus*, strawberry, persimmon, red bayberry, date palm, and kiwifruit [49,64,83,111,154,175]) or subsequent translocations to the SDR (e.g., *Ceratodon* [65]). Though not all the sex-determining genes in these systems show evidence of a recent duplication (e.g., *Asparagus TDF1* [49]). In these latter cases, genes with tissue-specific or narrower expression may be more likely to evolve a sex-determining role.

Although several different genes have evolved to be sex-determining, in other dioecious species where they remain autosomal, they often instead show sex-biased expression, suggesting they play a conserved, sex-specific role or may be regulated by the sex-determining (or other sex-linked) genes [192]. For example, in kiwifruit, *FrBy* is the Y-linked, male-fertility gene, but *TDF1* also shows male-biased expression [176], which makes sense given its role in tapetum development [50,193,194]. One pattern shared across many of these systems is the role many of these genes play in the cytokinin pathway (e.g., poplar, willow, date palm, and kiwifruit [69,83,88,175]), which is involved in floral development, particularly in the carpel and female gametophyte (reviewed in [195]). As we characterize the SDRs of more independent evolutions of dioecy, we will gain more insight on what genes are more likely to be involved, if any.

Another notable pattern emerging is the empirical support for the two-gene model for dioecy. In asparagus, kiwifruit, date palm, and grape [50,83,84,176], the SDRs have two genes involved in female and in male sterility (Figure 1). In-depth analyses in asparagus and kiwifruit have verified the Y-linked genes *TDF1* and *FrBy*, respectively, promote male development and *SOFF* and *SyGl*, respectively, suppress female development [50,176,177]. In date palm, *LOG-like* is a strong candidate for being the female-sterility gene and both *CYP703* and *GPAT3* are candidates for promoting male development, whereas in grapes, *VviYABBY3* and *VviAPT3* are both candidates for female-sterility genes and *VviINP1* for promoting male development. Though interestingly, in grapes, recombination between the tightly linked SDR on the X and the Y caused the transition back to hermaphroditism seen in domestic grapes [84]. The copy of *VviYABBY3* found in hermaphroditic individuals more-closely resembles the female haplotype, rather than the male Y-linked copy, adding some additional weight to *VviYABBY3* being the female-sterility gene [84]. It will be interesting if similar patterns of occasional recombination are involved in other transitions back to hermaphroditism (e.g., papaya) or if other processes like whole-genome duplications are involved [150]. In other systems, a single gene has been shown to be a sex-determining switch, like *ARR17* in poplar and *OGI* in persimmon [88,171]. Though this result does not dispute the two-gene model, as the putatively ancestral hermaphroditic population had to first transition to gyno- or androdioecious [146,196].

Recent genome assemblies in dioecious plants have revealed more than sex-determining genes. Some studies have uncovered similar patterns in the evolution of the SDR that have been found in animal systems. The ancestral origins of sex chromosomes in the bryophytes more-closely resemble that of mammalian, bird, and some insect lineages [65,197,198,199], where the evolution happened early in the clade and remains shared among most taxa. This contrasts with most angiosperm sex chromosomes, which have more recent and independent origins (see examples above), though once they evolve many are also stable (e.g., *Phoenix* [83]). In other genera there is clear evidence of turnovers [82,88,145,154], where the sex-determining gene translocates to a new autosome, similar to what has been found in frogs and some fishes [200]. Neo-sex chromosomes have also been found [65,106,201], which can involve either part or an entire autosomal chromosome fusing to one or both sex chromosomes. Some theories have been developed on why some sex chromosomes are conserved while others turnover (reviewed in [200]) and closer examinations across plants may provide new insights.

Other similar patterns of gene gain to the sex chromosomes have been found between plant and animal sex chromosomes. Key to the movement of genes from the pseudoautosomal region to the SDR is suppressed recombination, a classic signature of which is evolutionary strata [202]. These strata occur when suites of genes are added to the region of suppressed recombination at the same time, causing them to have similar levels of divergence between gametologs (measured in synonymous substitutions, *Ks*). Multiple recombination-suppression events thus show a stepwise pattern of *Ks* along the SDR, with lower *Ks* for more-recent captures and higher *Ks* for older-captured genes. Indeed, evidence of evolutionary strata has been found in several plant species [69,87,109], suggesting plants may experience similar selective pressures that drive gene gain as animals [203]. The structural changes that cause suppressed recombination have classically been shown to be chromosomal inversions and several recent papers have found evidence in plants to also support this [109,204]. Some other striking convergent patterns, like palindromes, have been found in animal and plant sex chromosomes [94,147]. These palindromes consist of large inverted repeats and genes within these regions can undergo conversion [94,147]. Together, these results highlight that there are many dynamic patterns in sex chromosomes that are shared across these kingdoms.

However, there are just as many differences as there are similarities that have been found. Most often seen to date are differences in how suppressed recombination occurs. In some species, suppressed recombination can evolve before the SDR [106] with several evolving in close proximity to centromeres [53,66]. In other systems hemizygosity between the SDR caused by a deletion on the X suppresses recombination, rather than other structural changes like inversions [50,83,146]. In others suppressed recombination can occur without any structural changes at all, likely through the build-up of transposable elements (TEs) [69]. This latter result contradicts the often-thought pattern that TEs will build up after recombination has been suppressed on the SDR. Indeed, other characteristic patterns of degeneration and gene loss thought to affect sex-specific chromosomes, or at the very least the tempo of these processes, are questioned in several recent analyses [65,87,203]. Much of this is likely due to haploid gene expression [205], which occurs in gametophyte stages in plants and exposes genes to purifying selection. Together these results beg the question of whether the proposed linear model for the stages of sex chromosome evolution is overall applicable to plants or if these systems represent interesting exceptions to otherwise encompassing rules (see also [206]).

## 5. The Future of Plant Sex Chromosome Research

Combined, plants provide many independent tests for the evolution of sex chromosomes. While here we have focused on land plants, algae also provide other exciting, independent evolutions [15,207]. Although, despite the many recent publications, we have only just begun to uncover what plant sex chromosomes can illuminate. Assuming 5% of the 300,000 species of angiosperms are dioecious (using conservative numbers), only ~0.3% of these species have had their genomes sequenced to date, with an order of magnitude fewer in the other major clades (Table 1). Thus, one clear path moving forward is to increase the number and phylogenetic breadth of high-quality genome assemblies and annotations of dioecious species. While this has traditionally meant assembling a single exemplar genome for a species, the future of sex chromosome genomics should encompass pangenomes [208] that incorporate within-species variation, as well as closely-related, non-dioecious sister taxa that serve as outgroups. As sequencing technologies continue to improve, and the costs decrease, this becomes more tractable. Adding gene co-expression analyses will uncover downstream regulatory pathways [173,209] and whether these are more conserved than the sex-determining genes [192]. In addition to gene annotations, we should move to consistently annotate non-coding sequences, like small RNAs, [88,171,210] and uncover their targets to better understand their role in sex-specific development and sex determination. Moreover, as technologies like CRISPR improve, and protocols are established for more species, functional validations of these results will likely become standard [211]. These discoveries are all valuable for breeding programs of dioecious and closely related hermaphroditic crops. In fact, most of the species described in this review are economically important. There are also applications for controlling invasive species, like in palmer amaranth [108]. Additionally, from a conservation perspective, focusing on dioecious species is especially pressing, as the sexes often respond to stressors differently, meaning that due to climate change these species may be especially at risk for extinction [212].

In addition to comparative and functional genomics, a lot more interdisciplinary work in dioecy and sex chromosome research awaits. We need to focus on many classic (albeit also constantly improving) analyses rather than just the so-called “cutting-edge”. We need to fund more field work to identify new, potentially dioecious species and common-garden analyses to characterize development (e.g., [213]). We need better-supported, species-level phylogenies to infer the number of evolutions of dioecy, for example using Angiosperm353 [214] and GoFlag (Genealogy of Flagellate plants) [215] probe sets. We need more cytological analyses, to uncover how these chromosomes behave in the cell (e.g., [216,217]) or verifying in what tissues genes are expressed (e.g., [50]). Together, through these many approaches, we can discover a wealth of untapped knowledge to better understand the rules at play in these complex and dynamic regions of the genome in plants [15].

## Figures and Tables

**Table 1:** Published dioecious nuclear genomes. The species listed here are dioecious, though for many others, closely related hermaphroditic or monoecious references may be available.

Lineage	Family	Species	Sex Chromosome Type	Citation
Moss	Ditrichaceae	*Ceratodon purpureus*	UV	[65]
Moss	Pottiaceae	*Syntrichia princeps*	UV	[71]
Moss	Fontinalaceae	*Fontinalis antipyretica*	UV	[72]
Moss	Hylocomiaceae	*Pleurozium schreberi*	UV	[73]
Liverwort	Marchantiaceae	*Marchantia polymorpha*	UV	[74,75]
Liverwort	Marchantiaceae	*Marchantia inflexa*	UV	[76]
Gymnosperm	Ginkgoaceae	*Ginkgo biloba*	XY	[77,78]
Gymnosperm	Gnetaceae	*Gnetum montanum*	Possibly XY	[79]
Angiosperm	Amborellaceae	*Amborella trichopoda*	ZW	[80]
Angiosperm	Dioscoreaceae	*Dioscorea alata*	XY	[81]
Angiosperm	Dioscoreaceae	*Dioscorea rotundata*	ZW	[82]
Angiosperm	Asparagaceae	*Asparagus officinalis*	XY	[49,50]
Angiosperm	Arecaceae	*Phoenix dactylifera*	XY	[83]
Angiosperm	Vitaceae	*Vitis arizonica*	XY	[84]
Angiosperm	Vitaceae	*Vitis amurensis*	XY	[85]
Angiosperm	Vitaceae	*Vitis riparia*	XY	[86]
Angiosperm	Vitaceae	*Vitis vinifera sylvestris*	XY	[84]
Angiosperm	Vitaceae	*Muscadinia rotundifolia*	XY	[84]
Angiosperm	Euphorbiaceae	*Mercurialis annua*	XY	[87]
Angiosperm	Salicaceae	*Populus alba*	ZW	[88]
Angiosperm	Salicaceae	*Populus deltoides*	XY	[88]
Angiosperm	Salicaceae	*Populus euphratica*	XY	[89]
Angiosperm	Salicaceae	*Populus ilicifolia*	XY	[90]
Angiosperm	Salicaceae	*Populus tremula*	XY	[88]
Angiosperm	Salicaceae	*Populus trichocarpa*	XY	[91]
Angiosperm	Salicaceae	*Salix brachista*	Possibly ZW	[92]
Angiosperm	Salicaceae	*Salix matsudana*	Possibly ZW	[93]
Angiosperm	Salicaceae	*Salix purpurea*	ZW	[94]
Angiosperm	Salicaceae	*Salix suchowensis*	ZW	[95]
Angiosperm	Salicaceae	*Salix viminalis*	ZW	[69]
Angiosperm	Rosaceae	*Fragaria x ananassa*	ZW	[96]
Angiosperm	Moraceae	*Ficus carica*	XY	[97]
Angiosperm	Moraceae	*Ficus erecta*	Possibly XY	[98]
Angiosperm	Moraceae	*Ficus hispida*	XY	[99]
Angiosperm	Cannabaceae	*Cannabis sativa*	XY	[100]
Angiosperm	Cannabaceae	*Humulus lupulus*	XY	[101]
Angiosperm	Myricaceae	*Morella rubra*	ZW	[64]
Angiosperm	Myricaceae	*Morus alba*	XY	[102]
Angiosperm	Myricaceae	*Morus notabilis*	Possibly XY	[103]
Angiosperm	Anacardiaceae	*Pistacia vera*	ZW	[104]
Angiosperm	Caricaceae	*Carica papaya*	XY	[54,105]
Angiosperm	Polygonaceae	*Rumex hastatulus*	XY	[106]
Angiosperm	Amaranthaceae	*Amaranthus palmeri*	XY	[107,108]
Angiosperm	Amaranthaceae	*Amaranthus tuberculatus*	XY	[108]
Angiosperm	Amaranthaceae	*Spinacia oleracea*	XY	[109]
Angiosperm	Simmondsiaceae	*Simmondsia chinensis*	XY	[110]
Angiosperm	Ebenaceae	*Diospyros lotus*	XY	[111]
Angiosperm	Actinidiaceae	*Actinidia chinensis*	XY	[112]
Angiosperm	Actinidiaceae	*Actinidia eriantha*	XY	[113]
Angiosperm	Solanaceae	*Solanum appendiculatum*	XY	[114]

## Data Availability

Not applicable.
